# Seroepidemiological Investigation of *Toxocariasis* in the Isparta Region of Turkey

**Published:** 2010-06

**Authors:** M Demirci, S Kaya, ES Çetin, BC Arıdoğan, S Önal, M Korkmaz

**Affiliations:** 1 Department of Clinical Microbiology, Health Minister, İzmir Atatürk Education and Research Hospital, Izmir, Turkey; 2 Department of Microbiology and Clinical Microbiology, Faculty of Medicine, Suleyman Demirel University, Isparta, Turkey; 3 Department of Parasitology, Faculty of Medicine, Ege University, Izmir, Turkey

**Keywords:** Toxocariasis, Seroprevalence, Risk factors, Turkey

## Abstract

**Background:**

Toxocariasis is a common disease around the world. Our objective was to determine *Toxocara* seroprevalence in humans in the city of Isparta, Southwest Turkey, in respect of some determinants such as age, socio-economic level, residence in city center or rural area etc.

**Methods:**

Five hundred and thirty four individual participants from Isparta center and 85 from Asagi Gokdere village were included in the study. *T. cati* specific antibodies were analyzed using excretory-secretory (ES)-enzyme-linked immunosorbent assay (ELISA) method.

**Results:**

*T. cati* antibodies were detected as positive in 73 (13.6%) of 534 samples which were collected from subjects living in the city center and 24 (28.2%) of 85 samples from Asagi Gokdere village. *Toxocara* seropositivity was detected among 15.6% of whole study group. The seroprevalence of toxocariasis was significantly higher among subjects from village than in subjects from city center (*P*=0.001). While gender, high school education, source of the water which is used, family income and geophagia/eating nail behaviors were the features which were detected as being associated with toxocariasis seropositivity (odds ratios=0.5; 6.52; 3.61; 0.43; 0.13 respectively), owning dogs or cats and hand washing were detected as being not associated with toxocariasis seropositivity (*P*>0.05). Furthermore, *Toxocara* seropositivity was significantly higher among subjects in 0–10 than >40 year-old group (*P*=0.02).

**Conclusion:**

It can be suggested that untreated lost pet population, environmental contamination, and way of life have influence on the epidemiology of toxocariasis.

## Introduction

The ascarids *Toxocara canis* and *T. cati* are the most widely distributed nematode parasites in canids as final hosts, and in many vertebrates including man as paratenic hosts. Soil contaminated with *T. canis* and *T. cati* embryonated eggs is the main source of infection of man (1, 2). Toxocariasis may appear in three following forms as ocular larva migrans (OLM), visceral larva migrans (VLM) and covert toxocariasis with nonspecific symptomatology. These forms can be seen with different various sings, symptoms and organ involvement. Although there is some evidence of tissue damage due to migrating or arrested larvae, most clinical symptoms are thought to be due to immune-mediated inflammatory responses (1, 3).

The laboratory diagnosis of human *Toxocara* infection is usually based on detection of antibodies against excretory/secretory antigen of this ascarid, using ELISA, which is easy to carry out, has good sensitivity and specificity, and shows good reproductivity (4–6). The use of serological assays has permitted the identification of the cosmopolitan characteristic of human *Toxocara* infection. The seroprevalence of toxocariasis has been reported as various proportions in humans from different regions of the world depending on the demographic composition of the population sampled as well as way of life and standard of living (7–9).

In this study, we aimed to determine the seroprevalence of toxocariasis in humans in the city of Isparta, Southwest Turkey, in respect of some determinants like age, socio-economic level, residence in city center or rural area etc.

## Materials and Methods

### Subjects

We undertook a multi-stage sampling analysis of data from Isparta, between May and August 2005. The sample size that should be taken to ensure a 95% confidence interval was considered 753 subjects, assuming the prevalence of toxocariasis as 2% and an error rate of 1%. Five hundred and thirty four individual participants (68.9% female; 31.1% male, mean age 28±20 yr) who were living in Isparta city center, at the Southwest region of Turkey and 85 participants (72.9% female; mean age 38.5± 20 yr) from Asagi Gokdere village of Isparta were included in the study. One hundred thirty-four samples were excluded from the study for various reasons, such as hemolysis, loss of sample sera, absent questionnaire and so forth. A total of 619 samples were included in the study. Eighteen health centers in Isparta city center were separated into regions according to their population number. The health centers were separated into groups as follows: 15–20 thousand population (Yedisehitler with 3 health centers); 10–15 thousand (Gülistan with 3 health centers); 5–10 thousand (Kurtulus and Karaagac with 6 health centers) and <5 thousand (Yenice and Cunur with 6 health centers). The population percentages were as follows: 35% from Yedisehitler, 25% from Gulistan, 18% from Kurtulus, 8% from Yenice, 4% from Cunur region. A questionnaire was constituted for the subjects, which included demographic features such as age, sex, occupation, animal ownership and exposures, diet, water sources, and type of sewage disposal. Blood samples were taken from the study group by venipuncture into polystyrene tubes. Sera were separated and stored at −20°C until used. The design of this study was reviewed and approved by the Ethical Committee of Süleyman Demirel University.

### Diagnostic methodology

All cases were examined for antibodies against *Toxocara* by modified ELISA employing as the excretory secretory antigen (ESA) product of the *Toxocara* (ES-ELISA) according to Glickman et al. (4), and Korkmaz (6). Briefly, *Toxocara* larvae were incubated at 37°C for one week in RPMI 1640 medium (Sigma) containing 100 U/mL penicilin and 250 µg/mL streptomycin (Pfizer, Istanbul, Turkey). The suspension containing ESA of *Toxocara* was centrifuged at 4°C (13,000xG) for two hours and was filtered by a 0.2−µm pore-size filter. ESA were coated to immunoplate (Costar, Schiphol Rijk, The Netherlands) at concentrations of 95 µg/mL. Human sera were used at 1:100 dilutions, and alkaline phosphatase-conjugated anti-human IgG (Sigma) was used at 1:10,000 dilutions. The substrate was 4-nitrophenyl phosphate disodium salt (Merck, Darmstadt, Germany). Plates were read on a microplate reader (Bio-Tek, ultramicroplate reader EL X 808, Winooski, USA) at an absorbance of 405 nm. Test serum, antigen, and conjugate titrations were determined with checkerboard titration. The cut-off value was calculated as the average of the absorbance values of negative sera +3 SD.

### Statistical methodology

The statistical method used to show the differences between the groups were Fisher's Exact test and a *P*-value less than 0.05 was considered statistically significant.

## Results

We detected *Toxocara* seropositivity in 97 of all study groups (15.7%). *Toxocara* antibodies were detected as positive in 73 (13.6%) of 534 subjects from city center and 24 (28.2%) of 85 subjects from AşağI Gökdere village. The seroprevalence of toxocariasis was higher among subjects from village than in subjects from city center and this difference was statistically significant (*P*=0.001, odds ratios=0.4). In addition to this, there were statistically significant difference between low socioeconomic city district (Cünür and Yenice, 24.6%) and high socioeconomic city district (Kurtulus and Gulistan, 9.2%) (*P*=0.002, odds ratios=3.2) (Table 1).

**Table 1:** Distribution of *Toxocara* seropositivity according to health centers

		Village (n=85)	Yedisehitler (n=187)	Gulistan (n=133)	Kurtulus (n=96)	Karaağaç (n=53)	Yenice (n=43)	Çünür (n=22)	Total n=619
*Toxocara* positivity	n (%)	24 (28.2)	28 (15.0)	13 (9.8)	8 (8.3)	8 (15.1)	10 (23.3)	6 (27.3)	97 (15.7)

While gender, high school education, source of the water which is used, family income and geophagia/eating nail behaviors were the features which were detected as being associated with toxocariasis seropositivity (odds ratios= 0.5; 6.52; 3.61; 0.43; 0.13 respectively), owning dogs or cats and hand washing were detected as being not associated with toxocariasis seropositivity (*P*> 0.05). Furthermore, *Toxocara* seropositivity was significantly higher among subjects in 0–10 year old group than >40 year-old group (*P*=0.02, odds ratios=0.7) although there was no significant difference between other age groups (*P*>0.05) (Table 2).

**Table 2:** *Toxocara* seropositivity and characteristics of study subjects in the Isparta region

		n	*Toxocara* Seropositivity n (%)	Statistic
Sex	Female	(430)	77 (17.9)	*P*=0.02
	Male	(189)	20 (10.6)	
Ages	0–10	(172)	31 (18.0)	*P*>0.05
	11–20	(78)	*13 (16.6)	
	21–40	(184)	28 (15.2)	*P=0.024
	40–more	(185)	25 (13.5)*	
Domicile	Village	(85)	24 (28.2)	*P*=0.001
	Centrum	(534)	73 (13.6)	
High school education	Yes	(65)	2 (3)	*P*=0.001
	No	(554)	95 (17.1)	
Source of the water	Network	(545)	71 (13)	*P*<0.001
which is used	No–network	(74)	26 (35.1)	
Hand washing	Yes	(499)	75 (15)	*P*>0.05
behaviour before meals	No	(120)	22 (18.3)	
Dog/cat ownership	Yes	(49)	10 (20.4)	*P*>0.05
	No	(570)	87 (15.2)	
Family income	Low	(204)	48 (23.5)	*P*<0.001
	High	(415)	49 (11.8)	
Geophagia/ eating nail	Yes	(48)	25 (52)	*P*<0.001
	No	(571)	72 (14.4)	

## Discussion

Our data show that *Toxocara* seropositivity is an important health problem in Isparta region of Turkey. However, insufficient attention is still being paid to this disease. Although serologic tests are useful tools in diagnosis of toxocariasis with particular asymptomatic or indistinguishable clinical manifestations they are not routinely used, so most of the toxocariasis cases are under-diagnosed in clinical practice. ES ELISA has been reported to have 78.3% specificity and 92.% sensitivity in the diagnosis of toxocariasis (4, 5, 10). However, there has been concern about cross-reactivity with other helminth infections. The low-cost easy-to-use ELISA method has therefore been recommended as a first-line test. As in many other infectious diseases, serologic tests in toxocariasis are important, when clinical signs are present. It is known that if a few eggs of *Toxocara* are ingested, seropositivity may develop without any clinical signs. Seropositivity may also be detected in cases with chronic infection without any clinical signs (5, 10, 11).

*Toxocara* has a worldwide distribution, both in developing and in industrialized countries. In our country, Korkmaz indicated the seropositivity of toxocariasis as 39.4%, 44.9%, 28.5% and 33.3% in suspected toxocariasis patients, allergic patients, healthy children, and healthy adults, respectively in the Izmir region (6). On the other hand, Kaplan et al. (12) reported lower seroprevalence rate (2.1%) in the ElazIg region. Oguztürk and Saygi (13) found seropositivity rate as 32.3% in the village schoolchildren in the Sivas region. In the Eskisehir region, *Toxocara* seroprevalence was detected among randomly selected 430 children in the rural areas as 16.9% and 0.7% of 141 children in the urban areas (14). While significant levels of anti-*Toxocara* antibodies were detected in rural area (16.9%), only one child (0.71%) had positive levels of anti- *Toxocara* antibodies from urban area. In the Istanbul region, Büyükbaba et al. (15) reported that the seropositivity rate was 42.2% in the rural area and 11.9% in the urban area. Total seroprevalence rate of *Toxocara* antibodies was found 12.9% in both groups. We concluded that toxocariasis might be considered as an important health problem also in our region, since seropositivity of toxocariasis, which we have determined in this study, was considerably high or same as reported in previous studies.

It is reported that *Toxocara* seroprevalence is variable in the world connected with some risk factors as well as favorable climate, presence of no untreated dogs and cats specially related with lost population, life of way related with soil, geophagia (direct or indirect), socioeconomic status related with hygiene and education levels (1, 2, 8, 16, 17). Humid and warm climates are probably the best ecosystems for embryonation and survival of *Toxocara* eggs in the soil (16). It was reported that the harsh climatic conditions existing in Argentina's Patagonia would inhibit embryonation of eggs in the soil, thus lowering the transmission of human toxocariasis (18). Although it is reported that *Toxocara* seroprevalence is high in those owning dogs and cats, it is accepted that the risk factor for toxocariasis is contact with soil contaminated with cat/dog faeces, rather than owning a cat or dog at home (17). Direct contact with infected dogs and cats plays a secondary role in transmission because of the egg of *Toxocara* required extrinsic incubation period in the soil before eggs become infective (1). Our results support these opinions because there was no significant difference between the two groups with regard to pet ownership. A pilot study, in the Lebanon, *Toxocara* seroprevalence, male gender, below high school education, higher numbers of persons in the household, and low family income during childhood, were significant (19).

The presence of dogs and cats in the public urban areas is common in many regions and several reports notified that fecal contamination meaningfully increases the risk of human infection by protozoa and helminth (8, 9, 20–22). The contamination of parks and gardens with helminth eggs and other pathogens may constitute a menace, at least in urban areas, to public health in the city that cats defecates parks. It is reported that fecal contamination of the environment in the central areas was higher than in suburbs in Italy (23). The explanation of this condition might be based on the numbers of pet owners residing in the center usually take animals to parks and gardens in the neighborhood specifically for the purpose of defecation. Contrary of this situation, in our region, in the cities dog and cat ownership ratio is less than European countries, thus association of dogs and cats with parks is limited but lost population dogs and cats are common especially in public areas in the villages. This might be the reason why we determined high seropositivity in the rural areas.

Children have far more chances to contaminate themselves than adults when playing in parks or on playground do. Particularly, habits such as pica, practiced by 10–30% of children between 1 and 6 years of age, is clearly associated with the probability of acquired parasitic infections (9, 24). Toxocariasis is seen more frequently among children than among adults due to such reasons as frequent contact with contaminated soil, poor hygiene, and consuming contaminated food (1, 8). In the India, gender was found a significant risk factor for the *Toxocara* infection in children population. Male children were found more infected (41.97% as compared to females (20.94%) (25). The risk factors that were found associated with the infection of toxocariasis in children population of Kashmir valley included family background, status of living conditions, awareness (25). In our study, we determined the highest seropositivity in children younger than 10 years in respect of adults, but no significant differences among other age groups. However, there were significant differences between the two groups with regard to geophagia/eating nail.

Socio-economic level is a factor that influences *Toxocara* seroprevalence (1, 2). While some studies report that *Toxocara* seroprevalence increases with low socio-economic status (19, 25–27), there are others which claim that it does not change (20, 28). This contradiction can be explained with the presence of different socio-economic factors influencing the seroprevalence of toxocariasis like dog and cat ownership, presence of untreated lost pet population, personal and social hygiene, education, fecal contamination of drinking water, soil, and park in the city and urban area. In the current study, it has been seen that there was difference between *Toxocara* seropositivity in terms of socio-economic level sign such as high school education, family income, and source of the water, which is used. This can be explained with the factors like environmental contamination and presence of untreated dogs and cats, which are mostly being seen in low socio-economic district than high socio-economic district. The combination of favorable climate and poor sanitation results in a high transmission pressure.

Finally, this study provides considerable information about toxocariasis in this region and sets the ground for a broader population-based epidemiological survey in the future to validate these results. In addition to this, more complete studies consisting of a routine test and a subsequent control of higher sensitivity techniques should be done in humans. Detailed information about seroprevalence and risk factors for toxocariasis will let us better realize and overcome this disease. On the other hand, it should not be underestimated that one feature which constitutes an important risk factor for a region or country may not be the most important risk factor for another region. It is likely that, toxocariasis will be one of the diseases about which we will be writing and talking in the future as long as human live with dogs and cats.
